# An Unusual Presentation of Erythema Multiforme Following the Administration of Pfizer-BioNTech COVID-19 mRNA Vaccine in a Pediatric Patient

**DOI:** 10.7759/cureus.58450

**Published:** 2024-04-17

**Authors:** Yara Alghamdi, Fahad Abdulghani, Hassan F Huwait, Magdy Abdulghani, Sahal J Samarkandy

**Affiliations:** 1 Dermatology, King Abdullah Medical Complex, Ministry of Health, Jeddah, SAU; 2 Dermatology, King Fahad Armed Forces Hospital, Jeddah, SAU; 3 Dermatopathology, Umm Al-Qura University, Jeddah, SAU; 4 College of Medicine, King Saud bin Abdulaziz University for Health Sciences, Jeddah, SAU; 5 Dermatology, King Abdulaziz Medical City (KAMC), Jeddah, SAU

**Keywords:** erythema multiforme minor, erythema multiforme, pfizer-biontech bnt162b2, bnt162b2 mrna vaccine, bnt162b2, pfizer-biontech covid-19 vaccine related adverse events, pfizer biontech covid-19 vaccine, pfizer, covid-19, dermatology

## Abstract

Coronavirus disease 2019 (COVID-19) caused a global calamity that forced emergency use authorization to Pfizer-BioNTech COVID-19 (BNT162b2) vaccine. It is efficacious in preventing symptomatic severe acute respiratory syndrome coronavirus-2 (SARS-CoV-2) infection in seronegative recipients. The safety profile is still unclear; however, commonly reported symptoms post-vaccination are fatigue, headache, muscle pain, chills, and injection-site pain. COVID-19 disease elicits, to some extent, cutaneous side effects like urticaria, morbilliform rash, and chilblain-like eruption. Vaccination against COVID-19 was reported to induce similar dermatologic manifestations, such as urticarial rash, delayed large-local reaction, local injection-site reaction, and morbilliform eruption. Erythema multiforme (EM) is a rare manifestation post-vaccination, and only a few reports implicate it as a culprit in cutaneous eruptions following the BNT162b2 vaccine. This report delineates the presentation of a healthy 14-year-old girl to a dermatology clinic who developed EM post-vaccination with the first dose of BNT162b2. New-onset EM-eruption post-vaccination with BNT162b2 had been reported previously in 14 cases, and one case reported on the flare of preexisting-EM.

## Introduction

Pfizer-BioNTech mRNA COVID-19 (BNT162b2) has undergone authorization to be administered to the public to prevent Coronavirus disease 2019 (COVID-19) [1]. It is administered to persons older than 12 years for the prevention of symptomatic severe acute respiratory syndrome coronavirus-2 (SARS-CoV-2) infection. It is an mRNA vaccine encoding SARS-CoV-2 spike-protein in lipid nanoparticles. Frequently reported side effects are miscellaneous and related to the immune system's reaction to vaccine components, like fatigue and muscular pain. The phenomenon of cutaneous eruption is of a peculiar manner. COVID-19 disease itself elicits dermatologic eruptions like urticaria, morbilliform rash, and chilblain-like reactions [1]. Nevertheless, vaccination against COVID-19 induces similar cutaneous manifestations, specifically, urticarial rash, delayed large-local reaction, local injection-site reaction, morbilliform eruption, erythromelalgia, and cosmetic-filler reaction [1]. Erythema multiforme (EM) is a rare dermatologic manifestation post-vaccination, and only 15 papers reported its eruption following the BNT162b2 vaccine [2-12]. Herein, a healthy adolescent female developed atypical EM after first-dose vaccination with BNT162b2.

## Case presentation

**Figure 1:**
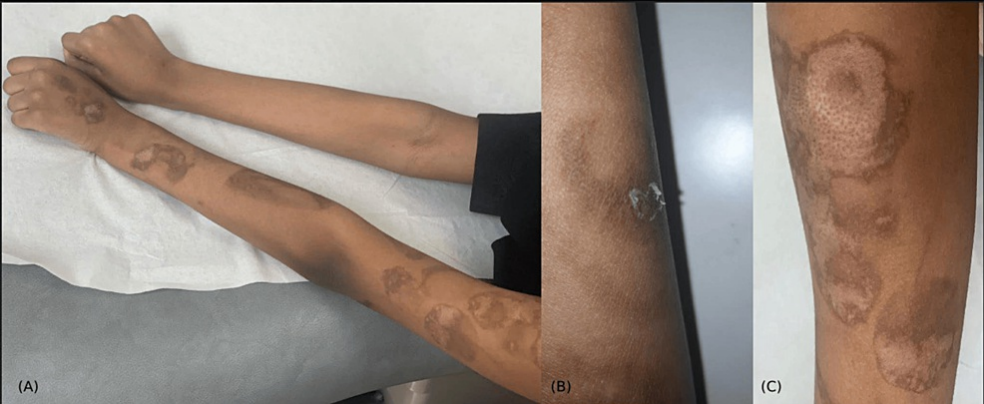
(A) Acute localized cutaneous eruption after the BNT162b2 vaccination over the left upper extremity. The eruption materialized firstly on the injection site, deltoid area, and disseminated downward through the arm and hand favoring the extensor surface. (B) Scale formation on the ill-defined incomplete ring-shaped dusky-tanned two-zoned targetoid plaque. (C) Two-zoned dusky purpuric center surrounded by a pale targetoid plaque over the extensor surface of the left arm.

**Figure 2:**
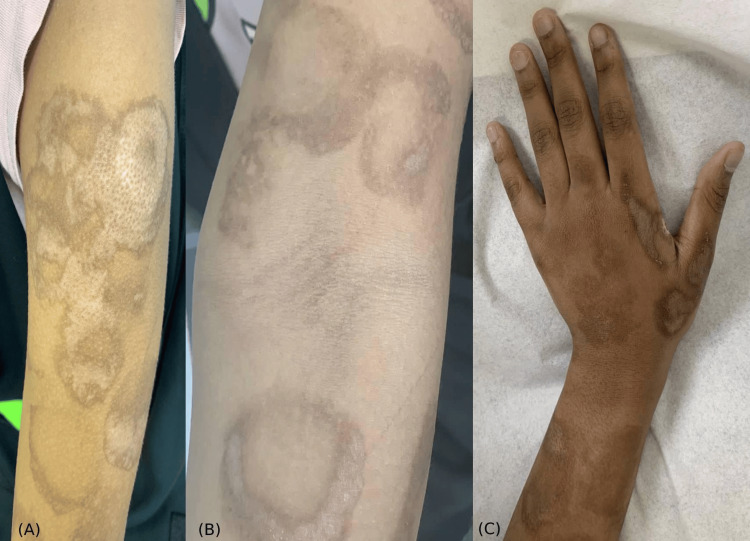
(A) Poorly defined morphed targetoid plaques with signs of PIH over the left deltoid area. (B) Incomplete ring-formation of targetoid lichenoid plaques with signs of PIH, xerosis, and excoriation on the anterior aspect of the arm. (C) Targetoid plaques taking the shape of incomplete rings with signs of post-inflammatory hyperpigmentation on the dorsal aspect of the hand. PIH: post-inflammatory hyperpigmentation

**Figure 3:**
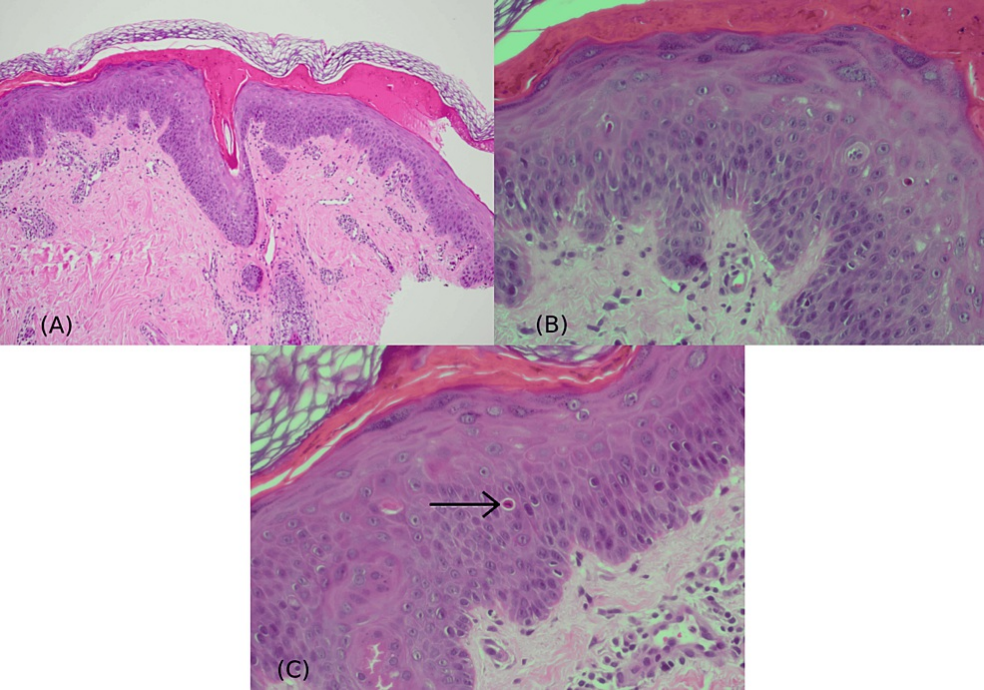
(A) Hematoxylin and eosin (H&E)-stained section shows orthokeratotic hyperkeratosis, overlying a layer of necrotic epidermis (x100 magnification). (B and C) The underlying epidermis is regenerative with predominantly scattered supra-basal dyskeratotic keratinocytes (arrow) (H&E; x40 magnification, features suggestive of EM pattern). EM: erythema multiforme

A healthy 14-year-old female developed localized unilateral cutaneous eruption throughout the left upper extremity that manifested after the administration of the BNT162b2 vaccine. Approximately one-hour post-vaccination, a localized fluid-filled bullous formed at the injection site. The next day, the bullous confluent into pruritic dusky-red two-zoned incomplete-ring targetoid scaly plaques (Figures 1-2). There was no face, ocular, mucosal, or systemic manifestation. New medications, previous history of similar lesions, signs of herpes-simplex-virus (HSV) infection, and prodromal symptoms were all denied. Referral for dermatological assessment was done 20 days post-eruption, and demonstrated ill-defined two-zoned scales with dusky-red incomplete-ring plaques over the left extremity, favoring the extensor surface (Figures 1-2). Histopathology showed a regenerative epidermis with scattered dyskeratotic keratinocytes (Figure 3). From a clinical and histopathological aspect, atypical EM secondary to the BNT162b2 vaccine was established. Usual management was offered with betamethasone-valerate 0.1% cream and fusidic-acid 2% cream to counteract any secondary bacterial infection. At the follow-up examination, EM-eruption had improved. Presentation of acute localized EM within the first four hours post-vaccination will hinder the patient from receiving the second dose of BNT162b2 and other mRNA vaccines.

## Discussion

EM is a self-limited immune-mediated reaction with unknown pathogenesis. The most frequent trigger is HSV [13]. It has been linked to SARS-CoV-2 infection as typical acral eruption in young persons, and widespread atypical manifestation in adults [4]. EM is a rare dermatologic manifestation post-vaccination, and only 15 papers reported on its eruption following the BNT162b2 vaccine [2-12].

One hypothesis that explains the abrupt onset of EM is pre-sensitization. Post-vaccination adverse events are generally attributed to the immune system's reaction to the vaccine’s components like egg protein, gelatin, and polyethylene-glycol (PEG). Those ingredients are necessary to stabilize vaccines during transportation, prevent bacterial contamination, and improve the drug’s water solubility; the latter is achieved by PEG [14]. BNT162b2 vaccine does not contain any food or drug components; however, it is formulated with PEG to stabilize lipid nanoparticles containing active SARS-CoV-2 mRNA-protein [14]. PEG is found in numerous medications and injectable steroids. Although safe, reports have shown that 70% of patients receiving PEG therapies will develop anti-PEG IgG [14,15]. Thus, reaction to PEG-formulated products (i.e. vaccines) indicates previous sensitization.

Vaccine-induced EM is highly infrequent. Nonetheless, it has been implicated before in literature with diphtheria-pertussis-tetanus, measles-mumps-rubella, and human papillomavirus vaccines [2]. Su et al. [16] have collected data from 1999 to 2017 and concluded the median time of EM onset post-vaccination is six days; while adverse events occurred within two weeks. The temporal association of the BNT162b2 vaccine and EM eruption in this report is unusual compared with the data from Su et al. [16] as it appeared within 24 hours.

EM is secondary to the BNT162b2 vaccine, although rare, but scarce reports exist [2-12]. The body of evidence from the literature reported 14 new cases, and one report described a flare of pre-existing EM [2-12]. The manifestation was highly variable from aspects of age, gender, dose-inflicted eruption, local or systemic manifestation, and anatomical involvement (Table 1). All reports described eruption in adults [2-12], there was only one adolescent report of EM [12]. The only consistent factor that supported EM diagnosis is the clinical and histopathological correlation. Almost all eruptions have manifested within two weeks, mostly in females (80%), after first-dose administration (66.66%), and resolved with topical corticosteroid.

**Table 1:** The table outlines the presentation and histological data of EM after the BNT162b2 vaccine Almost all eruptions have manifested within two weeks, mostly in females, and after first-dose administration. Anatomical distribution has favored acral sites, but older patients had more of a generalized eruption. Most cases reported no mucosal involvement. There was no previous HSV infection except in one case that described a flare of preexisting EM. All reports described EM eruption in adults, there was no pediatric report. All cases were managed conservatively, except two cases that needed hospital admission due to the severity of the lesions [4,9]. The only consistent factor that supported EM diagnosis among those reported cases is clinical and histopathological correlation. HSV^1^: Herpes simplex virus; EM^2^: erythema multiforme; BNT162b2^3^: Pfizer-BioNTech COVID-19 vaccine; DEJ^4^: dermoepidermal junction

Sources	Age	Gender	Dose	Anatomical distribution	Mucosal involvement	Temporal eruption	Histopathology report	Previous HSV^1^ infection
New-onset EM^2^ after BNT162b2^3^ Vaccine								
Borg et al. [8]	38	Male	First dose	Arm, elbow, and sole	Yes	2 days post-vaccination	Perivascular lymphocytic and histiocytic infiltrate in the upper dermis. Apoptotic keratinocytes throughout epidermis.	No
Kim et al. [2]	78	Female	First dose	Generalized	Yes	10 days after vaccination	Necrotic keratinocytes and subepidermal bullae with lymphocytic and eosinophilic infiltrate in DEJ^4^.	No
Sechi et al. [3]	76	Female	First dose	Acral distribution	-	4 days after vaccination	Vacuolar interface dermatitis with inflammatory lymphohistiocytic infiltrate in dermis. Mild epidermal spongiosis.	No
Bonino et al. [4]	91	Female	Second dose	Neck, trunk, back, and extremities	No	6 days after vaccination	Perivascular lymphocytic infiltrate in DEJ^4^. Dyskeratotic keratinocytes in the basal layer. Satellite lymphocytes.	­­No
Wunderlich and Dirschka [5]	61	Female	Second dose	Acral distribution	Yes	2 days after vaccination	Necrotic keratinocytes in epidermis obscuring DEJ^4^. Papillary edema. Perivascular inflammatory infiltrate, and eosinophilia.	No
de Las Vecillas et al. [6]	47	Female	Second dose	Generalized	No	1 day after vaccination	Interstitial perivascular dermatitis. Lymphohistiocytic and eosinophilic infiltrate. Intraepidermal and subcorneal spongiotic vesicles.	No
Scharf et al. [7]	27	Female	First dose	Acral distribution (also observed on the patient's melanocytic nevi)	-	3 days after vaccination	No histology was obtained. Diagnosis of Nevocentric EM^2^ was made on clinical grounds.	No
Charfi et al. [10]	51	Female	First dose	Acral distribution	-	5 days after vaccination	No histology was obtained. Diagnosis of EM^2^ was made on clinical grounds.	No
	55	Male	Second dose (EM^2^ also appeared after first dose)	Upper and lower extremities	-	6 days after vaccination	No histology was obtained. Diagnosis of EM^2^ was made on clinical grounds.	No
Katayama and Ota [11]	60	Female	Second dose	Elbows	No	3 days after vaccination	Keratinocytes apoptosis, with basal vacuolar change, spongiosis with lymphocytes, and perivascular lymphohistiocytic infiltrate.	No
Petruzzi et al. [12]	55	Female	First dose (EM^2^ also flared after second dose)	Oral, genital, knees, and acral involvement	Yes	1 day after vaccination	No histology was obtained. Diagnosis of EM^2^ was made on clinical grounds.	No
	49	Female	Second dose	Oral (tongue, gingiva, buccal mucosa, mouth floor, and soft palate)	Yes	1 day after vaccination	No histology was obtained. Diagnosis of EM^2^ was made on clinical grounds.	No
	20	Female	First dose	Oral and genital involvement	Yes	18 days after vaccination	No histology was obtained. Diagnosis of EM^2^ was made on clinical grounds.	No
	15	Male	First dose	Generalized	Yes	7 days after vaccination	No histology was obtained. Diagnosis of EM^2^ was made on clinical grounds.	No
EM flare-up after BNT162b2 Vaccine								
Livery et al. [9]	58	Female	First dose (similar eruption has occurred 24 hours after second dose vaccination with BNT162b2^3^)	Acral distribution (bilateral palms and soles)	No	12 hours after vaccination	-	Yes (Herpes labialis)

## Conclusions

EM pathogenesis, theoretically, involves a stimulus triggering a delayed hypersensitivity reaction. This stimulus could be PEG, and previous sensitization could explain the rapid manifestation in this report. EM secondary to the BNT162b2 vaccine is unusual. Nonetheless, the clinical picture along with histopathological correlation is suggestive of EM.
